# Prevalence and Correlates of Unintentional Injuries among In-School Adolescents in Ghana

**DOI:** 10.3390/ijerph18136800

**Published:** 2021-06-24

**Authors:** Richard Gyan Aboagye, Abdul-Aziz Seidu, Samuel Adolf Bosoka, John Elvis Hagan, Bright Opoku Ahinkorah

**Affiliations:** 1 Department of Family and Community Health, School of Public Health, University of Health and Allied Sciences, Hohoe PMB 31, Ghana; raboagye18@sph.uhas.edu.gh; 2 Department of Population and Health, University of Cape Coast, Cape Coast PMB TF0494, Ghana; abdul-aziz.seidu@stu.ucc.edu.gh; 3 College of Public Health, Medical and Veterinary Sciences, James Cook University, Townsville, QLD 4811, Australia; 4 Department of Estate Management, Takoradi Technical University, Takoradi P.O. Box 256, Ghana; 5 Department of Epidemiology and Biostatistics, School of Public Health, University of Health and Allied Sciences, Hohoe PMB 31, Ghana; samuelbosoka@gmail.com; 6 Physical Education and Recreation, Department of Health, University of Cape Coast, Cape Coast PMB TF0494, Ghana; 7 Neurocognition and Action-Biomechanics-Research Group, Faculty of Psychology and Sports Science, Bielefeld University, Postfach 100131, 33501 Bielefeld, Germany; 8 School of Public Health, Faculty of Health, University of Technology Sydney, Sydney, NSW 2007, Australia; brightahinkorah@gmail.com

**Keywords:** adolescents, Ghana, injury, psychological distress, psychosocial, substance use

## Abstract

Injuries among adolescents pose significant public health problems. Unintentional injuries are the leading cause of adolescents’ mortality and disability with the largest burden in low-and middle-income countries. Yet, there is paucity of data in Ghana on adolescent injuries. The present study aimed to determine the prevalence and correlates of unintentional injuries among in-school adolescents in Ghana using data from the Global School-Based Health Survey. Cross-sectional data on 2058 adolescents in junior and senior high schools who randomly participated in the 2012 Global School-Based Health Survey were analyzed. Descriptive statistics were performed to determine the prevalence of unintentional injuriesacross the background characteristics of in-school adolescents. Binary logistic regression was employed to determine the factors associated with unintentional injuries. The results were presented as crude and adjusted odds ratios at a 95% confidence interval. The prevalence of one or more serious injuries in the past 12 months was 57.0%. The most commonly reported type and cause of injuries were “I had a cut or stab wound” (15.2%) and “I fell” (13.1%), respectively. In the adjusted regression, in-school adolescents aged 14–16 (aOR = 1.60, CI = 1.12–2.28) were more likely to report one or more serious injuries compared to their counterparts aged 13 or younger. In-school adolescents who participated in physical education (aOR = 1.27, CI = 1.03–1.58) had higher odds of reporting one or more serious injuries. The odds of being injured was higher among adolescents who were truant at school compared to those who were not truant (aOR = 1.42, CI = 1.14–1.77) In-school adolescents who were bullied were more likely to report being injured one or multiple times compared to their counterparts who were not bullied (aOR = 2.16, CI = 1.75–2.65). In addition, the odds of being injured once or multiple times were higher among adolescents who were physically attacked (aOR = 2.21, CI = 1.78–2.75), those that engaged in physical fighting (aOR = 1.94, CI = 1.54–2.45), and those who reported high psychological distress (aOR = 2.00, CI = 1.52–2.63) compared to their counterparts who were not. Conversely, adolescents in senior high schools were 39% less likely to be injured once or multiple times compared to those in junior high schools (aOR = 0.61, CI = 0.47–0.79). A relatively high prevalence of unintentional injuries was found among in-school adolescents in the study. The numerous factors identified in this study could be integrated into health promotion and injury prevention activities to help reduce the occurrence of injuries among in-school adolescents. Moreover, students who are susceptible to unintended injuries such as older adolescents, victims of bullying, those who participate in physical education, those who are often involved in fights, truants, and those who have psychological distress should be sensitized to take measures that will reduce their level of susceptibility. First aid treatment services should also be made available in schools to treat victims of unintended injuries.

## 1. Introduction

Childhood injuries have emerged as significant public health concerns necessitating urgent attention [1,2,3]. Adolescent serious injury can be defined as any form of injury that can make an adolescent miss usual activities or an injury that requires medical attention [1]. In 2016, injuries accounted for more than 255 million disability-adjusted life years (DALYs) [4]. Globally, approximately 2300 children and adolescents die every day from unintentional injuries [5]. These injuries consist of road traffic injuries, drowning, poisoning, falls, and burns [3], with the former alone causing an estimated 10.2 deaths per 100,000 adolescents [5]. Road traffic injuries were the leading cause of adolescents’ mortality in 2016 with over 135,000 deaths [6,7], while nearly 50,000 adolescents died from drowning [7]. As a result, unintentional injuries are regarded as the leading cause of death and disability among adolescents [7]. In addition to the mortalities, injuries are associated with a lifelong disability, psychosocial problems, and financial repercussions on the injured victim and their families [3,8,9]. However, this burden is disproportionately distributed, with the largest problem in low- and middle-income countries, impacting adversely on the health and productivity, and the resulting pressure on social systems [8,10].

A study conducted among adolescents aged 12–15 years from 68 low- and middle-income countries reported an overall 42.9% prevalence of serious injuries in the past 12 months [1]. A study by Beck et al. [11] found the prevalence of one or more serious injuries among adolescents from Argentina, Uruguay Chile, and Bolivia to be 27.1%, 29.5%, 30.9%, and36.8% respectively. In seven Caribbean countries, the percentage of adolescents reporting one or more serious injuries within the past 12 months was 54.3% for all countries, ranging from 43.1% in Dominica to 59.5% in Jamaica [12]. Additionally, the prevalence of serious injury in the past year was 43.1% in the Cook Islands, 40.8% in Niue, 73.8% in Samoa, and 49.1% in Tonga [13].

In sub-Saharan Africa, a study conducted among adolescents in six countries showed an overall 68.2% prevalence of one or more serious injuries of which country-specific rates were Zambia (71.5%), Kenya (71.0%), Uganda (63.4%), Zimbabwe (62.8%), Namibia (60.2%), and Swaziland (38.6%), [14]. Similarly, findings from other studies using Global School-Based Health Survey (GSHS) data reported the prevalence of serious injuries in the following countriesMozambique (55.7%) [15], Djibouti (61.1%) [2], Botswana (65.8%) [16], and Tanzania (22.14%) [17].

Evidence suggests that factors such as sociodemographic characteristics (male sex and low socioeconomic status) [14,17,18,19,20,21,22] substance use (alcohol, tobacco, smoking, drugs) [2,14,15,18,20,21,23,24,25,26], soft drinks consumption, physical education at school [15,20,22], psychological distress [15,20,22,23], being bullied [2,18,19,23], engaging in physical fight [2,18,19,25], truancy [14,20,22,25], ever had sex [20,21], depression [14], loneliness [14,24,25], and parental or guardian support and bonding [20,22] are associated with unintentional injuries among adolescents.

In Ghana, there is a general dearth of literature on adolescents’ injuries. A retrospective descriptive study by Morna et al. [27] reported 17.6% of injury-related deaths from 2012–2018 among children aged 16 years and below at the Cape Coast Teaching Hospital (Cape Coast, Ghana). Another study conducted in the Korle-Bu Teaching Hospital (Accra, Ghana), showed that injury-related deaths accounted for 17% of all autopsies performed on adolescents (10–19 years) between 2001 and 2003 [28]. Road traffic injuries and drowning were the most reported cause of injury-related mortalities in both studies [27,28].

Due to the burden of unintentional injuries among adolescents, there is the need to understand the country-specific nature and distribution of these injuries to better inform educational campaigns, evidence-based planning, priority settings, and resource allocation for its prevention and control [1,29]. Hence, this study seeks to determine the prevalence and factors influencing unintentional injuries among in-school adolescents using nationally representative data.

## 2. Materials and Methods

### 2.1. Study Design and Sampling Technique

Data for this study were obtained from the 2012 Global School-Based Health Survey (GSHS) of Ghana [30]. The 2012 GSHS of Ghana was conducted in the country among adolescents with partnership from the World Health Organization (WHO), Center for Disease Control and Prevention (CDC, Atlanta, GA, USA), Middle Tennessee University (Murfreesboro, TN, USA), and Ghana Education Service (GES, Accra, Ghana). The survey data were collected using a cross-sectional study design from WHO countries. Close-ended structured questionnaires were used to collect the data from the students. The students self-reported their responses to each question on a computer scannable answer sheet. Generally, the GSHS survey measures behavioral risk and protective factors among adolescents which has a propensity of causing morbidity and mortality in youths and adults. These factors include; alcohol and other drug use, dietary behaviors, hygiene, mental health, physical activity, protective factors, sexual behavior, tobacco use, violence, and unintentional injury [31].

The study participants were recruited from junior and senior high schools in all the administrative regions in Ghana (then 10 regions). A two-stage cluster sampling technique was used in selecting the schools and students, respectively. At the initial stage, the study schools were selected with probability proportional to the school’s enrolment size. At the last stage, classes within chosen schools were randomly selected, and all the students in the sampled classrooms were eligible to participate in the survey. However, the survey included students who were aged 10 to 19 years (period of adolescence), present at school on the day of data collection, and showed evidence of written informed consent (those aged 18 years and above), and written parental or guardian consent form and child assent form (those between 18 years). This sampling method ensured that every eligible student had an equal chance of being selected for inclusion in the study. Numerical weights were applied to each student’s record to enable the generalization of results to in-school adolescents. A total of 3632 students from junior and senior high schools participated in the survey. Of this, 1648 and 1984 students were from junior and senior high schools, respectively. Among the junior high schools (JHS), the school, student, and overall response rates were 100%, 82%, and 82%, respectively. Similarly, that of the senior high schools (SHS) was 96%, 74%, and 71%. However, 2058 students with complete cases on all selected variables were used as the sample for the current study. The dataset is available freely at https://www.who.int/ncds/surveillance/gshs/ghanadataset/en/ (accessed on 1 February2020). We relied on the “Strengthening the Reporting of Observational Studies in Epidemiology” (STROBE) statement in writing the manuscript [32].

### 2.2. Study Variables

#### 2.2.1. Outcome Variable

The main outcome variable was self-reported injury. This was assessed using the question “During the past 12 months, how many times were you seriously injured?” A total of eight responses were provided ranging from 1 = 0 times through to 8 = 12 or more times. A response of “0 times” means the absence of injury in the previous 12 months, while responses from 1 to 12 or more showed that the student had sustained one or more serious injuries. Serious injury was defined as when it makes you miss at least one full day of usual activities (such as school, sports, or a job) or requires treatment by a doctor or nurse. Additional two questions addressing the type and cause of serious injury were also assessed. Detailed questions, responses, and coding can be found in Table 1.

#### 2.2.2. Explanatory Variables

Independent variables used to determine the predictors of one or more serious injuries were sociodemographic characteristics (age, sex, grade), hunger (a proxy to socio-economic status), substance use (current alcohol use, smoking cigarettes, tobacco use, marijuana use, drugs use), peer support, psychological distress, and parental or guardian support and bonding. The explanatory variables were selected based on findings from previous studies [20,21,22,23,33]. Psychological distress was assessed using five items (loneliness, anxiety, suicidal ideation, suicidal attempt, and close friends). All five items were summed. The summed items were grouped into “0 = 0” low; “1 = 1” medium and “2–5” = 3 high. The same process was repeated for parental or guardian support and bonding. An index was created by summing all four items. The index created was grouped into “0–1” = low; “2” = medium; and “3–4” = high. Psychological distress and parental or guardian support and bonding were classified per previous similar studies [20,22,23]. The specific variables, sample questions, responses, and coding can be found in Table 1.

### 2.3. Statistical Analyses

Data analyses were carried out using Stata software version 16.0 (Stata Corporation, College Station, TX, USA). The dataset was cleaned and recoded before the final analysis. Missing data on the variables of interest were not included in the analysis. All the recoded variables in this study were referred to other studies that used GSHS data [20,21,22,23,33]. Two-level analysis using descriptive and inferential statistics was performed. Firstly, descriptive statistics employing frequencies, percentages, and confidence intervals were done to determine the prevalence of injury, types of injury, and causes of injury. Lastly, binary logistic regression was used to determine the independent predictors of injury. Two binary logistic regression models (bivariate and multivariable) were built with the first model showing the independent association between each of the explanatory variables and the outcome variable, while the second model looked at the joint association between all the explanatory variables and injured once or multiple times. In the bivariate model, due to multiple-testing, we introduced a correction method by using the Bonferroni correction method. This was done by dividing the alpha rate (*p* = 0.05) by the number of analysis performed (17 explanatory variables). Thus, 0.05/17 = 0.0029. Therefore, at the bivariate analysis, statistical significance was declared at *p* < 0.003. However, to avoid the assumption that a variable that is not significant at the bivariate level will not be significant at the multivariable level, we included all variables in the multivariable model, irrespective of their significance at the bivariate level of the regression analysis. The results of the logistic regression were presented using crude and adjusted odds ratios and their respective 95% confidence intervals signifying the level of precision. We checked for multicollinearity with Variance Inflation Factor (VIF). We found a mean VIF of 1.52 showing that there was no evidence of multicollinearity among the variables. A *p*-value of less than 5% or 0.05 was considered significant. Complex sample analysis (svy) and the inherent sample weight were applied in all analyses to reduce bias from non-response and improve generalizability to all in-school adolescents in Ghana. The reference categories for all the explanatory variables were based on previous studies that used the GSHS dataset [20,21,22,23].

### 2.4. *Ethical Consideration*

Ethics approval was not required for this study since the data is secondary and is available in the public domain. However, the survey was approved by the Institutional Review Board at Middle Tennessee State University. Before the conduct of the survey, institutional permission to carry out the study was sought from the GES. All the GES’s ethical guidelines and policies concerning the use of students were strictly adhered to. Written informed consent to participate in the study was obtained from school managers and students aged 18 years and above. Parental consent and child assent were sought from students below 18 years. Students anonymously and voluntarily completed the questionnaire.

## 3. Results

### 3.1. Prevalence of Injury and Background Characteristics of Respondents

Of the 2058 in-school adolescents considered for the analysis, 56.3% were males. The majority (51.5%) were aged 17 years or older. Most (61.2%) of the students were in SHS. The overall prevalence of once or multiple times of serious injuries was 57.0% (55.7% among males and 58.7% among females). Over sixty-six percent of the students in JHS reported having been injured, while 63.1% of students aged 14–16 years were injured. Among those who reported having been injured once or multiple times, hunger, physical education, truancy, physical fighting, physical attack and peer support contributed to 63.9%, 73.8%, 67.9%, 72.8%, 75.9%, 75.4% and 53.4% of the prevalence, respectively. Current alcohol use (73.1%), current marijuana use (94.4%), smoking cigarette (84.1%), tobacco use (82.2%), drug use (91.0%), high psychological distress (74.3%), and high parental support (54.3%) were reported among students who were injured once or multiple times (see Table 2).

### 3.2. Prevalence of Injury Events among the Respondents

Regarding the injury events, the majority (15.2%) of the adolescents reported: “I had a cut or stab wound” as the leading type of injury, followed by a broken bone or dislocated joint (10.6%), concussion or head injury (3.7%), and “I had a burn” (2.4%). Adolescents who reported “I had a gunshot” and “I was poisoned” recorded 0.9% each. Boys reported slightly more types of serious injuries compared to girls in “I had a cut or stab wound”, a broken bone or dislocated joint, and “I had a gunshot”. Most reported causes of serious injury were “I fell” (13.1%), “something fell on me or hit me” (8.7%), motor vehicle accidents, “I was attacked” (2.9%), “Was in a fire” (2.4%), and “breathed something bad” (1.8%) (see Table 3).

### 3.3. Association between Injured Once or Multiple Times and Explanatory Variables

Table 4 shows the logistic regression model on the association between background characteristics and experience of injury (injured once or multiple times). At the adjusted stage, students aged 14–16 (aOR = 1.60, CI = 1.12–2.28); students who participated in physical education (aOR = 1.27, CI = 1.03–1.58); those who were truant at school (aOR = 1.42, CI = 1.14–1.77); those who were bullied (aOR = 2.16, CI = 1.75–2.65); being physically attacked (aOR = 2.21, CI = 1.78–2.75); those who engaged in physical fight (aOR = 1.94, CI = 1.54–2.45), and those with high psychological distress (aOR = 2.00, CI = 1.52–2.63) were more likely to be injured once or multiple times. Students from SHS were 39% less likely to be injured once or multiple times (aOR = 0.61, CI = 0.47–0.79) compared to those in JHS.

## 4. Discussion

This study sought to determine the prevalence and factors influencing unintentional injuries among in-school adolescents in Ghana. The prevalence of injury (injured once or multiple times) among adolescents in Ghana was 57.0%. This finding is similar to that of Mozambique [15] and Jamaica [12], which reported 55.7% and 59.5%, respectively. The present finding is higher than the overall prevalence from studies conducted in Malaysia [20], and among four Southeast Asian countries (Indonesia, Myanmar, Sri Lanka, and Thailand) [23]. All the studies reported prevalence rates below fifty percent. However, higher rates were found in Egypt (68.5%) [33] Djibouti (61.1%) [2], 65.8% in Botswana [16], and 70% in Timor-Leste [24]. Additionally, our finding on the prevalence of unintended injuries was lower compared to that of Pelzter [14], who reported a 68.2% prevalence of one or multiple injuries among adolescents from six African countries. Differences in school settings and external environmental factors could explain the observed differences in the prevalence rates.

In the current study, the most reported causes of serious injuries were; fall, hit by an object or something, and motor vehicle accidents, while the majority reported cut or stab wound, a broken bone or dislocated joint, and head injury or concussion as the frequent type of serious injury. Similar findings have been reported in other GSHS studies [13,15,20,22,23,24].

Adolescents aged 14–16 years were more likely to report serious injuries compared to the younger age group. Though unintentional injuries are prevalent across an individual’s lifespan, adolescents in this age group (14–16 years) could have encountered risks that might be different from other age groups [34]. Additionally, varied behavioral, physical, and cognitive abilities, coupled with curiosity and impulsivity, could account for the observed association [34]. A consistent finding was reported in a study conducted in Spain [35].

SHS students were less likely to report being injured once or multiple times. This finding concurs with a study conducted in China [36]. Another study by Albashtawy et al. [37] reported that students in the lower level, thus grade 7, were more likely to report being injured compared to those of higher levels. Adolescents in higher levels could be experiencing variations in growth and development stages and changes in their activities and playing patterns, which could have contributed to low injury prevalence among them [37]. Additionally, poor individual safety awareness and adventure and impulsiveness inherent in younger students with poor self-restraint could explain the observed association in the present study [36].

Being truant at school was found to increase the odds of injury among adolescents. Consistent findings have been reported by other researchers using GSHS data in Egypt [33], Malaysia [20], and Botswana [16]. A possible explanation for the observed association could be that truant adolescents are more likely to engage in behaviors (such as violence, illegal road behaviors, drugs, and alcohol use) that contribute to the likelihood of an injury [24].

Consistent with other studies conducted among adolescents [15,20,22,26], the present study reported that participating in physical education at school was associated with higher odds of one or multiple injuries. As explained by Carmeli et al. [38], lack of sufficient warm-up, poor playing grounds, unsuitable outfits, lack of safety precautions, and high extraneous exercises that have a high mechanical impact could account for the reported injuries among students who partake in physical education.

Adolescent aggressive behaviors: bullying, physical fight, and physical attack were all associated with higher odds of serious injury in the present study. Researchers posited that an adolescent exhibiting one form of aggressive behavior could be a response or reaction to other violent conduct towards him or her. For instance, Jansen et al. [19] stipulated that being a victim or perpetrator of bullying is associated with a greater tendency to engage in a physical fight, which can cause an injury that requires medical treatment. Other studies in the Philippines and five ASEAN countries reported that adolescents who were bullied were more likely to engage in a physical fight [39,40]. Another study among adolescents in seven Caribbean countries concluded that being in a physical fight was associated with risky behaviors such as being bullied [41]. The association between being bullied, engaged in a physical fight, and being physically attacked and injury occurrence have been reported by scholars from other studies [11,13,14,18,19,20].

The study found high psychological distress as contributing to the increase in the likelihood of injury among adolescents. Psychological distress may have poorly affected the adolescents’ physical and mental health making them susceptible to undertaking risky behaviors with injury being the most likely consequence [13]. The association in the present study is in line with those of previous studies [15,20,22].

### Limitations of the Study

The cross-sectional nature of the dataset used is not appropriate to establish any causal inferences from the results obtained. Recall bias is another limitation. The self-reported nature of the questions could be subjected to social desirability and non-response biases. The study involved only in-schooladolescents. Hence, the findings cannot be generalized to the entire adolescent population. The exclusion of missing cases from the dataset before analysis led to the reduction in sample size which could have impacted on the results of the study. Additionally, the analysis was based on the risk factors found in the dataset, while other potential factors that can influence injury occurrence were not assessed. Additionally, there was possible underreporting of injuries as data were not collected from students who were absent on the day of data collection. The data used for the analysis was nine years old and does not present the current situation of unintended injuries among in-school adolescents in Ghana. However, the findings of our paper can guide in developing current interventions to deal with unintended injuries among in-school adolescents in the country.

## 5. Conclusions

A relatively high prevalence of injury occurrence was found in the present study. Factors that were associated with an increased likelihood of injury among the adolescents were being aged 14–16 years, being bullied, participating in physical education at school, being involved in a physical fight, being truant at school, being physically attacked, and having high psychological distress. Adolescents from SHS were less likely to be injured. There is a need for collaborative and integrative health promotion and injury prevention programs and interventions to help curb this menace. Moreover, students who are susceptible to unintended injuries such as older adolescents, victims of bullying, those who participate in physical education, those who often involve in fights, truants, and those who have psychological distress should be sensitized to take measures that will reduce their level of susceptibility. First aid treatment services should also be made available in schools to treat victims of unintended injuries.

## Figures and Tables

**Table 1:** Study variables.

Variables	Question	Response Options and Recoding
**Outcome variable**		
Injury	During the past 12 months, how many times were you seriously injured?	1 = 0 times; to 8 = 12 or more time **(coded as 1 = 0; 2–8 = 1)**
**Injury events**		
Type of injury	During the past 12 months, what was the most serious injury that happened to you?	I was not seriously injured during the past 12 months I had a broken bone or a dislocated joint I had a cut or stab wound I had a concussion or other head or neck injury was knocked out, or could not breathe I had a gunshot wound I had a bad burn I was poisoned or took too much of a drug Something else happened to me
Cause of injury	During the past 12 months, what was the major cause of the most serious injury that happened to you?	I was not seriously injured during the past 12 months I was in a motor vehicle accident or hit by a motor vehicle I fell Something fell on me or hit me I was attacked or abused or was fighting with someone I was in a fire or too near a flame or something hot I inhaled or swallowed something bad for me Something else caused my injury
**Explanatory variables**		
Age	How old are you?	1 = 11 years or younger; to 8 = 18 years or older **(coded as 1–3 = 0; 4–6 = 1; 7–8 = 2)**
Sex	What is your sex?	1 = male, 2 = female **(coded as 2 = 0; and 1 = 1)**
Grade	In what grade are you?	**Coded as JHS = 0; and SHS = 1**
Physical education	During this school year, on how many days did you go to physical education (PE) class each week?	1 = 0 days; to 6 = 5 or more days **(coded 1 = 0 and2–6 = 1)**
Truancy	During the past 30 days, on how many days did you miss classes or school without permission?	1 = 0 days; to 5= 10 or more **(coded 1 = 0 and 2–5 = 1)**
Hunger (a proxy of socioeconomic status)	During the past 30 days, how often did you go hungry because there was no enough food in your home?	1 = never; to 5 = always **(coded 1–3 = 0, 4–5 = 1)**
Bullied	During the past 30 days, on how many days were you bullied?	1 = 0 days; to 7 = All 30 days **(coded 1 = 0; 2–7 = 1)**
Physical fight	During the past 12 months, how many times were you in a physical fight?	1 = 0 times; to 8 = 12 or more times **(coded 1 = 0 and 2–8 = 1)**
Physically attacked	During the past 12 months, how many times were you physically attacked?	1 = 0 times; to 8 = 12 or more times **(coded 1 = 0 and 2–8 = 1)**
Peer support	During the past 30 days, how often were most of the students in your school kind and helpful?	1 = never; to 5 = always **(coded 1–3 = 0 and 4–5 = 1)**
**Substance use**		
Current alcohol use	During the past 30 days, on how many days did you have at least one drink containing alcohol?	1 = 0 days; to 7 = All 30 days **(coded as 1 = 0; 2–7 = 1)**
Current tobacco use	During the past 30 days, on how many days did you use any tobacco products other than cigarettes, such as tawa snuff powder, chewing tobacco, paper rolled tobacco, dip, cigar, or pipe?	1 = 0 days; to 7 = All 30 days **(coded as 1 = 0 and 2–7 = 1)**
Current cigarettes use (current smoking)	During the past 30 days, how many days did you smoke cigarettes?	1 = 0 days; to 7 = All 30 days **(coded as 1 = 0 and 2–7 = 1)**
Current marijuana use	During the past 30 days, how many times have you used marijuana (also called wee, Jah, Indian hemp, ahabammono, and ganja)?	1 = 0 times; to 5 = 20 or more times **(coded as 1 = 0 and 2–5 = 1)**
Drug use	During your life, how many times have you used amphetamines or methamphetamines (also called ice or yellow)?	1 = 0 times; to 5 = 20 or more times **(coded as 1 = 0 and 2–5 = 1)**
**Psychological distress**		
Loneliness	During the past 12 months, how often have you felt lonely?	1 = never, 2 = rarely, 3 = sometimes, 4 = most of the time to 5 = always **(coded as 1–3 = 0 and 4–5 = 1)**
Anxiety	During the past 12 months, how often have you been so worried about something that you could not sleep at night?	1 = never to 5 = always **(coded 1–3 = 0 and 4–5 = 1)**
Suicide attempt	During the past 12 months, how many times did you actually attempt suicide?	1 = yes, 2 = no **(coded as 2 = 0; and 1 = 1)**
Close friends	How many close friends do you have?	1 = 0; to 4 = 3 or more **(coded as 1 = 1; and 2–4 = 0)**
**Parental or guardian support**		
Parental supervision	During the past 30 days, how often did your parents or guardians check to see if your homework was done?	1 = never; to 5 = always **(coded 1–3 = 0; 4–5 = 1)**
Parental Connectedness	During the past 30 days, how often did your parents or guardians understand your problems and worries?	1 = never; to 5 = always **(coded 1–3 = 0; 4–5 = 1)**
Parental or guardian Bonding	During the past 30 days, how often did your parents or guardians really know what you were doing with your free time?	1 = never; to 5 = always **(coded 1–3 = 0; 4–5 = 1)**
Parental respect for privacy	During the past 30 days, how often did your parents or guardians go through your things without your approval?	1 = never; to 5 = alwayss **(coded 1–3 = 0 and 4–5 = 2)**

**Table 2:** Sample background characteristics and prevalence of injuries.

Injured Once or Multiple Times *n* = 2058				
Variable	Frequency	Percentage	Yes (%)	No (%)
**Injured Once or Multiple Times**			**57.0**	**43.0**
**Sex**				
Female	900	43.7	58.7	41.3
Male	1158	56.3	55.7	44.2
**Age**				
13 years or younger	240	11.7	62.5	37.5
14–16 years	758	36.8	63.1	36.9
17 years or older	1060	51.5	51.5	48.5
**School**				
JHS	798	38.8	66.8	33.2
SHS	1260	61.2	50.9	49.1
**Hunger**				
No	1806	87.8	56.1	43.9
Yes	252	12.2	63.9	36.1
**Physical education**				
No	604	29.3	66.5	33.5
Yes	1454	70.7	73.8	26.2
**Truancy**				
No	1363	66.2	51.5	48.5
Yes	695	33.8	67.9	32.1
**Bullied**				
No	1114	54.1	43.7	56.3
Yes	944	45.9	72.8	27.2
**Physical attack**				
No	1262	61.3	45.5	54.5
Yes	796	38.7	75.4	24.6
**Physical fight**				
No	1353	65.7	47.2	52.8
Yes	705	34.3	75.9	24.1
**Peer support**				
No	1374	66.8	58.9	41.1
Yes	684	33.2	53.4	46.6
**Alcohol use**				
No	1813	88.1	54.9	45.1
Yes	245	11.9	73.1	26.9
**Smoking cigarette**				
No	1970	95.7	55.8	44.2
Yes	88	4.3	84.1	15.9
**Marijuana use**				
No	1987	96.6	55.7	44.3
Yes	71	3.4	94.4	5.6
**Tobacco use**				
No	1923	93.4	55.3	44.7
Yes	135	6.6	82.2	17.8
**Drug use**				
No	1958	95.1	55.3	44.7
Yes	100	4.9	91.0	9.0
**Psychological distress**				
Low	1124	54.6	50.1	49.9
Medium	495	24.1	57.6	42.4
High	439	21.3	74.3	25.7
**Parental or guardian support**				
Low	1173	57.0	58.4	41.6
Medium	447	21.7	56.2	43.8
High	438	21.3	54.3	45.7

JHS = junior high school, SHS = senior high school.

**Table 3:** Prevalence of injury events by sex.

Variable	Total (%) (CI)	Boys (%) (CI)	Girls (%) (CI)
**Type of injury**			
Broken bone or dislocated joint	10.6 (9.3, 12.0)	11.6 (9.8, 13.6)	9.3 (7.5, 11.4)
I had a cut or stab wound	15.2 (13.7, 16.8)	16.8 (14.7, 19.1)	13.1 (11.0, 15.5)
Concussion/head injury	3.7 (2.9, 4.7)	3.1 (2.2, 4.3)	4.6 (3.3, 6.1)
I had a gunshot wound	0.9 (0.6, 1.4)	1.1 (0.6, 1.9)	0.7 (0.2, 1.4)
I had a burn	2.4 (1.8, 3.2)	1.6 (1.0, 2.6)	3.4 (2.4, 4.9)
I was poisoned	0.9 (0.5, 1.4)	0.7 (0.3, 1.4)	1.1 (0.5, 2.0)
Something else happened to me	11.7 (10.3, 13.1)	9.4 (7.8, 11.2)	14.5 (12.3, 17.0)
**Cause of injury**			
Motor vehicle accidents	5.5 (4.5, 6.6)	7.0 (5.6, 8.6)	3.6 (2.4, 5.0)
I fell	13.1 (11.6, 14.6)	12.4 (10.6, 14.5)	13.9 (11.7, 16.3)
Something fell on me or hit me	8.7 (7.6, 10.1)	8.8 (7.2, 10.6)	8.7 (6.9, 10.7)
I was attacked	2.9 (2.2, 3.7)	2.8 (2.0, 4.0)	3.0 (2.0, 4.3)
Was in a fire	2.4 (1.8, 3.1)	1.6 (0.9, 2.4)	3.4 (2.4, 4.9)
Breathed something bad	1.8 (1.3, 2.5)	1.3 (0.7, 2.1)	2.4 (1.5, 3.7)
Something else	11.2 (9.8, 12.7)	10.0 (8.3, 11.9)	12.8 (10.7, 15.1)

CI = Confidence Interval.

**Table 4:** Binary logistic regression analysis of predictors of unintentional injury.

	One or More Injuries	
Variable	COR (95% CI) *p*-Value	AOR (95% CI) *p*-Value
**Sex**		
Female	1.0	1.0
Male	0.89 (0.75, 1.06) 0.190	0.94 (0.77, 1.14) 0.525
**Age**		
13 years or younger	1.0	1.0
14–16 years	1.02 (0.76, 1.38) 0.875	1.60 (1.12, 2.28) 0.009
17 years or older	0.64 (0.48, 0.85) 0.002	1.43 (0.96, 2.13) 0.074
**School**		
JHS	1.0	1.0
SHS	0.51 (0.43, 0.62) <0.001	0.61 (0.47, 0.79) <0.001
**Hunger**		
No	1.0	1.0
Yes	1.38 (1.05, 1.82) 0.020	1.14 (0.83, 1.55) 0.416
**Physical education**		
No	1.0	1.0
Yes	1.42 (1.17, 1.71) <0.001	1.27 (1.03, 1.58) 0.026
**Truancy**		
No	1.0	1.0
Yes	1.99 (1.65, 2.41) <0.001	1.42 (1.14, 1.77) 0.002
**Bullied**		
No	1.0	1.0
Yes	3.44 (2.86, 4.14) <0.001	2.16 (1.75, 2.65) <0.001
**Physical attack**		
No	1.0	1.0
Yes	3.67 (3.02, 4.46) <0.001	2.21 (1.78, 2.75) <0.001
**Physical fight**		
No	1.0	1.0
Yes	3.52 (2.87, 4.31) <0.001	1.94 (1.54, 2.45) <0.001
**Peer support**		
No	1.0	1.0
Yes	0.80 (0.66, 0.96) 0.017	0.94 (0.75, 1.17) 0.568
**Current alcohol use**		
No	1.0	1.0
Yes	2.23 (1.66, 3.00) <0.001	1.11 (0.78, 1.58) 0.562
**Current smoking cigarette**		
No	1.0	1.0
Yes	4.18 (2.34, 7.45) <0.001	0.75 (0.35, 1.61) 0.466
**Current marijuana use**		
No	1.0	1.0
Yes	13.32 (4.84, 36.66) <0.001	3.08 (0.93, 10.26) 0.067
**Current tobacco use**		
No	1.0	1.0
Yes	3.74 (2.39, 5.87) <0.001	1.21 (0.69, 2.13) 0.501
**Drug use**		
No	1.0	1.0
Yes	8.17 (4.09, 16.30) <0.001	2.08 (0.93, 4.66) 0.074
**Psychological distress**		
Low	1.0	1.0
Medium	1.35 (1.09, 1.67) 0.006	1.14 (0.90, 1.45) 0.278
High	2.87 (2.25, 3.67) <0.001	2.00 (1.52, 2.63) <0.001
**Parental or guardian support**		
Low	1.0	1.0
Medium	0.91 (0.73, 1.14) 0.414	0.81 (0.71, 1.17) 0.477
High	0.84 (0.68, 1.06) 0.143	0.97 (0.75, 1.26) 0.850

AOR = Adjusted Odds Ratio; COR = Crude Odds Ratio; CI = Confidence Interval, 1.0 = reference category.

## Data Availability

The data for this study are available from WHO: https://www.who.int/ncds/surveillance/gshs/ghanadataset/en/ (accessed on 1 February 2020).
